# The dual role of tissue regulatory T cells in tissue repair: return to homeostasis or fibrosis

**DOI:** 10.3389/fimmu.2025.1560578

**Published:** 2025-03-06

**Authors:** Peiyan Zhang, Jiawei Wang, Jinlin Miao, Ping Zhu

**Affiliations:** Department of Clinical Immunology of Xijing Hospital and Department of Cell Biology of National Translational Science Center for Molecular Medicine, Fourth Military Medical University, Xi’an, Shaanxi, China

**Keywords:** tissue regulatory T cells, tissue repair, fibrosis, immune cells, non-immune cells

## Abstract

Tissue resident regulatory T cells (tissue Tregs) are vital for maintaining immune homeostasis and controlling inflammation. They aid in repairing damaged tissues and influencing the progression of fibrosis. However, despite extensive research on how tissue Tregs interact with immune and non-immune cells during tissue repair, their pro- and anti-fibrotic effects in chronic tissue injury remain unclear. Understanding how tissue Tregs interact with various cell types, as well as their roles in chronic injury and fibrosis, is crucial for uncovering the mechanisms behind these conditions. In this review, we describe the roles of tissue Tregs in repair and fibrosis across different tissues and explore potential strategies for regulating tissue homeostasis. These insights hold promise for providing new perspectives and approaches for the treatment of irreversible fibrotic diseases.

## 1 Introduction

Organ damage and fibrosis are common pathological processes observed in clinical settings, which are typically a consequence of inadequate healing following tissue injury (1). Fibrosis, characterized by an excessive activation of fibroblasts and collagen deposition that can ultimately lead to organ dysfunction (2), is closely linked to chronic inflammation, frequently marked by abnormal immune or non-immune cell activation and cytokine dysregulation (3, 4). Thus, understanding the mechanisms underlying tissue repair and fibrosis is essential for developing effective treatment strategies.

Regulatory T cells (Tregs) are essential immune system components that primarily maintain immune tolerance and tissue homeostasis (5). Tregs exhibit specific phenotypes in different types of tissue, thereby enabling them to sense and respond to changes in the local microenvironment (6), modulate inflammatory responses (7), and maintain tissue homeostasis *in situ* (8). The Tregs comprising this specialized subset are referred to as tissue Tregs (9, 10). In addition to their well-established immunosuppressive functions, they have recently been found to play pivotal roles in regulating tissue damage and regeneration (11).

Given the diversity and complexity of the regulatory mechanisms that modulate tissue damage repair, Tregs can play dual roles in both tissue repair and fibrosis. While they can suppress inflammatory responses and promote tissue repair (12), under certain conditions, activated tissue Tregs may also facilitate the progression of fibrosis (13). For example, by secreting amphiregulin (AREG), these Tregs can stimulate the proliferation of fibroblasts, which has been shown to exacerbate fibrotic processes (14). Conversely, studies have demonstrated that the absence of tissue Tregs in models of chronic liver injury worsens liver fibrosis, underscoring their protective role in the fibrosis process (15). Consequently, further studies examining the mechanisms of action of Tregs with respect to different types of tissue damage and fibrosis could provide valuable insights for developing targeted therapies.

## 2 Tissue Tregs

### 2.1 Basic characteristics of tissue Tregs

In addition to the expression of classical markers, such as CD4, CD25, and transcription factor forkhead box protein 3 (Foxp3) (16), tissue Tregs are also characterized by the expression of other functional molecules, which may differ according to tissue type. These Tregs not only display T cell receptors (TCRs) that recognize unique antigens but also characterized by tissue-specific transcriptional expression in addition to Foxp3 (including the *Il1rl1* gene encoding the IL-33 receptor ST2), which may make key contributions to their tissue-protective functions (17). The findings of recent research utilizing mouse single-cell sequencing data have indicated that tissue Tregs are characterized a common phenotype among different types of tissue, which features the prominent expression of markers typical of tissue-resident memory cells, such as CD69, CD103, CD11a, programmed cell death protein 1 (PD-1), and killer cell lectin-like receptor G1 (KLRG1) (18, 19). The expression of these activated functional molecules influences the residency of Tregs in tissues and facilitates their specific functions (20–22).

### 2.2 Mechanisms of tissue Tregs’ residency

The tissue homing ability of tissue Tregs is a fundamental aspect of their definition and function, influencing their distribution and roles across different tissues (23). The localization and homing of Tregs are influenced by various factors, include transcription factors, surface molecules, chemokines and their receptors, as well as lectins and their receptors. Additionally, numerous cytokines and signaling molecules in the tissue microenvironment play important roles in Tregs anchoring and homing (24–26).

#### 2.2.1 Transcription factors

Among transcription factors, Hobit and Blimp-1 are central to the regulation of tissue residency of lymphocytes, including Tregs (24). KLF2 (Kruppel-like factor 2) regulates the migration patterns of naïve Tregs by modulating homeostatic and inflammatory homing receptors. In the absence of KLF2, Tregs cannot effectively migrate to secondary lymphoid organs (SLOs), and this reduction in migration can trigger autoimmune diseases, underscoring that SLOs are critical for maintaining peripheral tolerance. The severity of the disease correlates with impaired recruitment of Tregs to SLOs, while enhancing the entry of Tregs into SLOs can alleviate autoimmune conditions. Furthermore, stabilizing KLF2 expression within Tregs can enhance peripheral tolerance, underscoring the importance roles of KLF2 in regulating the trafficking of Tregs to SLOs (27). Other transcription factors also significant impact the localization and migration of Tregs. For instance, RUNX1 and BAF60b are associated with CCR9 expression on Tregs, thereby affecting their migration to inflamed tissues. The activity of RUNX1 is closely related to the function and homing capabilities of Tregs, while BAF60b inhibits the inflammatory process by regulating Treg migratory capacity. BAF60b functions as a transcriptional coactivator that interacts with RUNX1 to enhance CCR9 expression on Tregs, which in turn affects their ability to migrate to inflamed tissues (28). Furthermore, FOXO1 is another transcription factor that plays a significant role in regulating homing molecule expression in Tregs. Activation of FOXO1 can enhance Treg responses to chemokines, thereby improving their localization ability in specific microenvironments (29).

#### 2.2.2 Chemokine receptors and adhesion molecules

The expression of specific chemokine receptors and adhesion molecules by Tregs allows them to respond to tissue chemokines, enabling precise migration and localization within those tissues (30). For instance, the expression of chemokine (C-C motif) receptor 4 (CCR4) enables Tregs to migrate to tissues such as the skin and lungs, where chemoattractant chemokine ligand (CCL) 17 and CCL22 are expressed (31, 32). CCR6 (33) and CCR10 (34) are highly expressed in intestine Tregs, promoting their homing and functional maintenance in the gut, Similarly, CCR9 binds to CCL25, facilitating the migration of Tregs to the intestine (35). G-protein-coupled receptor-15 (GPR15) also plays a critical role in regulating the homing of tissue Tregs to the colon (36). Mechanistically, the synergistic interaction between aryl hydrocarbon receptor (AhR) and Foxp3 enhances GPR15 expression in Tregs, whereas RORγt antagonizes AhR binding to the GPR15 site, thereby inhibiting GPR15 expression (37). Additionally, Tregs expressing high levels of CXCR4 preferentially home to the bone marrow, helping to alleviate inflammation (38). The expression of CCR5 is related to the migration potential of Tregs to inflammatory sites (39–41). Modulating the expression of these chemokines and activating their receptors can influence the homing and function of tissue Tregs, potentially providing therapeutic benefits in various immune-related diseases.

Adhesion molecules, such as intercellular adhesion molecule-1 (ICAM-1), also play a crucial role in the homing and tissue residency of Tregs (42). Lymphocyte Function-Associated Antigen-1 (LFA-1) is another key adhesion molecule that enhances Tregs adhesion to target cells or endothelial cells through interactions with ICAMs, thereby promoting their tissue-specific homing (43, 44). In mouse model, Tregs lacking LFA-1 exhibit significant homing defects, suggesting its indispensable role in Tregs migration (45). Furthermore, the α4β7 integrin expressed on Tregs facilitates their homing to the intestine by binding to mucosal addressin cell adhesion molecule-1 (MAdCAM-1) on intestinal endothelial cells (46). Layilin (LAYN), a C-type lectin-like receptor, is preferentially and highly expressed on activated Treg subsets in both healthy and diseased human skin. While LAYN expression on Tregs has minimal impact on the activation and *in vitro* suppressive capacity of Tregs, it exerts a cumulative anchoring effect on their dynamic movement *in vivo*. Specifically, LAYN promotes Tregs adhesion to the skin while restricting their suppressive capacity in the process (47).

#### 2.2.3 Tissue microenvironment

The microenvironment of different tissues features unique cellular composition, cytokines, and metabolic characteristics, all of which significantly influence the homing and functional regulation of tissue Tregs. Tregs not only express T cell receptors (TCRs) that recognize specific tissue antigens but also respond specifically to factors released following tissue damage (20, 48). For example, IL-33, a cytokine from the IL-1 family, acts as an “alarm” molecule during inflammation and tissue injury. Produced by epithelial and endothelial cells, IL-33 promotes the migration of ST2-expressing Tregs into tissues to suppress local inflammation (49, 50). Similarly, IL-18 facilitates Tregs migration to the thymus via CCR6-CCL20 interaction (51), while IL-35 enhances Treg migration and suppressive functions by upregulating CCR5 expression (52). Furthermore, IL-2 is essential for Treg development, function, and homing to the gut, skin, and inflammatory sites (53, 54).

The metabolic environment within tissue also influences Tregs residence, proliferation, and maintenance. For example, in the atherosclerosis microenvironment, oxidized phospholipids impair Tregs function and homing (55). Retinoic acid (RA) enhances the expression of receptors that guide Tregs to the gut (56). Dietary components like L-tryptophan have been shown to regulate Tregs numbers by affecting the transcriptional level of GPR15, thereby influencing Treg homing and local immune homeostasis (57). Dopamine, a key regulator of leukocyte migration, affects immune cell migration based on precise local concentrations. Low dopamine levels preferentially activate high-affinity dopamine receptors DRD3 in Tregs, weakening their suppressive capacity and limiting their recruitment into the gut mucosa (58). These studies suggest that the chemical components and biological signals in the microenvironment not only affect Treg survival and function but also directly impact their homing mechanisms, highlighting the critical role of the microenvironment in regulating the behavior of tissue Tregs.

In summary, the homing mechanisms of Tregs are complex processes involving multiple factors and interactions, including the regulation of various signaling pathways and cytokines. These mechanisms not only affect the homing ability of Tregs but may also alter their functional characteristics, playing a significant regulatory role in various immune-related diseases.

### 2.3 Functions of tissue Tregs

Tissue Tregs have been shown to contribute to the maintenance of tissue health via well-established anti-inflammatory mechanisms (7), including the secretion of anti-inflammatory cytokines such as interleukin 10 (IL-10) (59) and transforming growth factor β (TGF-β) (60), which suppress the activity of effector T cells (Teffs). Additionally, Tregs can directly eliminated Teffs via the release of granzymes and perforin (61). They also compete with Teffs for interleukin 2 (IL-2) (62), thereby reducing both the responsiveness of target cells and the availability of IL-2, and also produce extracellular enzymes such as CD39 and CD73 (63) that promote adenosine production and interfere with Teffs metabolism. Moreover, these Tregs induce tolerance in dendritic cells via inhibitory receptors that include lymphocyte-associated protein 4 (CTLA-4) (64) and lymphocyte activation gene 3 (LAG3) (65), thereby further suppressing the activity of Teffs (66). Collectively, these mechanisms contribute to the key roles played by Tregs in maintaining immune homeostasis and preventing the occurrence of autoimmune diseases.

The findings of recent studies have revealed that in addition to their immunosuppressive effects, tissue Tregs are also characterized by non-immune regulatory functions (67), including a wide range of effects regarding tissue repair (68), angiogenesis (69), basal metabolism (70), and maintenance of the stem cell niche (71). Depending on the specific type of tissue or model, tissue Tregs can either promote or inhibit angiogenesis (72), and have been shown to contribute to the unique stem cell niche in the skin (73, 74), bone marrow (75), and gut (76), and during pregnancy, facilitate vascular remodeling in the uterus (77). Tissue Tregs are well characterized in adipose tissues, particularly visceral adipose tissue, in which they play key roles in regulating insulin sensitivity and supporting lipid metabolism (78), and their roles in tissue repair have also been extensively documented (79, 80), primarily in muscles (81), lungs (82), skin (83), and the central nervous system (84). Moreover, these Tregs have been established to secrete AREG (85) and keratinocyte growth factor (KGF) (86), which are essential for the induction of epithelial cell proliferation in the lungs (87) and skin (88), as well as in muscle-associated satellite cells (81).

Tissue Tregs demonstrate context-dependent functional duality in disease progression. While growing evidence highlights their beneficial role in suppressing inflammation and promoting tissue regeneration, emerging studies reveal their paradoxical capacity to drive fibrosis in specific pathological settings. Therefore, a comprehensive investigation into the mechanisms by which Tregs influence tissue repair and fibrosis is essential for understanding their dual roles in different pathological contexts and for providing new insights for clinical treatment.

## 3 Interactions between tissue Tregs and immune/non-immune cells in tissue repair and fibrosis

The regulation of tissue Tregs during tissue repair after acute injury and chronic inflammatory responses involves the intricate interplay of tissue Tregs with various immune and non-immune cells (**Table 1**).

**Table 1:** The roles of tissue Tregs in tissue repair.

Tissue	Interacting cells (Immune/non-immune cells)	Mechanisms	Reference	Effect
Lung Tregs	Neutrophils	Inhibit the activation and chemotaxis of neutrophils	(89, 90) (91)	Inhibit inflammation
	Macrophages	Promote macrophages transform to M2 phenotype	(92)	Inhibit inflammation
		Secrete IL-13, promote the production of IL-10 by macrophages	(93)	Inhibit inflammation
	ILC2s	Suppress the activation of ILC2s	(94)	Inhibit inflammation
	γδT	TNFR2^+^Tregs suppress γδT-secreted pro-inflammatory IL-17A	(95)	Inhibit inflammation
		ST2^+^ Tregs enhance the expression of *Ebi3*	(96)	Inhibit inflammation
	Teffs	ST2^+^ Tregs secrete IL-13	(97)	Inhibit inflammation
		CD103^+^Tregs suppress Th2 responses via the high expression of IL-10	(98)	Inhibit inflammation
	Alveolar endothelial cells	Promote the proliferation of alveolar endothelial cells	(99)	Promote angiogenesis
	AT2	Promote the recovery of AT2 by increasing neutrophil infiltration and upregulating the release of TGF-β1	(100)	Promote regeneration
		Expression of KGF and AREG to regulate AT2 proliferation and differentiation	(86–88)	Promote regeneration
		Increase AT2 cell proliferation in a CD103-dependent manner	(101)	Promote regeneration
Skin Tregs	Innate cells	Reduce the accumulation of pro-inflammatory macrophages	(102)	Inhibit inflammation
		Utilize Jag1-Notch signaling to recruit innate cells	(103)	Promote repair
	Keratinocytes	Secrete AREG, drive keratinocyte proliferation	(104)	Promote repair
		Express PENK, promote the growth of epidermal keratinocytes	(105)	Promote repair
	Pericytes	Enhance pericyte TGF-β activation to restore vascular integrity	(104)	Promote repair
	HFSCs	Promote the differentiation of HFSCs to epithelial cells	(106)	Promote repair
Cardiac Tregs	Macrophages	Suppress M1, promote their transformation into M2	(107)	Inhibit inflammation
	Neutrophil	Promote Neutrophil apoptosis	(108)	Inhibit inflammation
	CMs	Inhibit the secretion of pro-inflammatory cytokines from CMs	(109)	Promote repair
		Reduce the apoptosis of CMs	(109)	Promote repair
		Promote CM proliferation	(109)	Promote repair
	ECs	Modulate the activation of ECs and influence angiogenesis	(110)	Promote repair
Muscle and Bone Tregs	Macrophages	Promote macrophages transform to M2 phenotype	(111)	Inhibit inflammation
	MPCs	Activate and expand MPCs	(112)	Promote repair
Intestinal Tregs	Epithelial stem cells	Support the renewal of epithelial stem cells	(113)	Promote repair
Brain Tregs	Astrocytes	Secrete AREG and neuron-specific genes to modulate astrocyte responses	(84)	Promote repair

ILC2s, type 2 innate lymphoid cells; TNFR, tumor necrosis factor receptor; ST2, growth stimulation expressed gene 2; Ebi3, Epstein-Barr virus-induced gene 3; Teffs: effector T cells; IL, interleukin; TGF-β, transforming growth factor-β; Th, T helper cells; AT2, alveolar type II cells; KGF, keratinocyte growth factor; AREG, amphiregulin; CD, cluster of differentiation; HFSCs, hair follicle stem cells; PENK, proenkephalin; CMs, cardiomyocytes; CCL, chemoattractant chemokine ligand; GAS: growth arrest-specific; ECs, endothelial cells; MPCs, muscle progenitor cells.

### 3.1 In acute tissue injury

The immune response triggered by acute injury is complex and involves interactions among various immune cells. Following injury, damaged tissues release a variety of cytokines and chemokines that attract immune cells, such as neutrophils, macrophages, and T cells, to the site of injury (114). While these cells clear dead cells and pathogens, they also release pro-inflammatory factors to promote tissue repair. However, excessive inflammatory responses can lead to further tissue damage, thereby affecting the repair process. Animal models lacking Tregs exhibit excessive inflammatory responses and impaired tissue repair, suggesting that Tregs play a crucial role in modulating inflammation during this process (115, 116). They maintain immune homeostasis by suppressing excessive inflammation, which creates favorable conditions for tissue regeneration and repair (117–120). For instance, Tregs can resolve LPS-induced lung inflammation and promote tissue repair by modulating T helper (Th)1 and Th17 responses (120). In models of acute injury to mouse bone, muscle, and skin, local delivery of Tregs has been shown to promote tissue repair and regeneration by reducing the accumulation of neutrophils and cytotoxic T cells that produce pro-inflammatory cytokine IFN-γ (11). In turn, these responses facilitate the transition of monocytes/macrophages (Mo/MΦ) to an anti-inflammatory and pro-healing state, thereby accelerating wound healing (11). Moreover, in mice with corneal alkali burns, subconjunctival injection of Tregs has been shown to reduce excessive inflammation by producing IL-10 and TGF-β, while also improving corneal healing by increasing AREG levels and activating epidermal growth factor receptor (EGFR) (121).

### 3.2 In chronic inflammatory responses

In the context of chronic injury or the chronic phase following acute inflammation, tissue Tregs affect the inflammatory response by interacting with immune cells and also promote tissue repair by influencing local non-immune cells (122), such as parenchymal cells and stem cells. For example, Tregs stimulate the growth of alveolar type II (AT2) cells in damaged lung tissue, accelerating wound healing and tissue regeneration (123). Co-culture experiments demonstrate that Tregs directly enhance the proliferation of AT2 cells in a CD103-dependent manner, as CD103 binds to E-cadherin expressed by epithelial cells (124). Furthermore, *in vivo* depletion of Tregs in the mouse lung injury model not only reduced AT2 cells proliferation but also delayed the recovery of lung injury, and similar effects are observed when blocking CD103 (124). In addition, Tregs play a crucial role in promoting regeneration through modulation of tissue stem cells. These stem cells can be rapidly activated after tissue damage, migrate to the injury site, and repair the damaged tissue by differentiating into specific cell types. Studies suggest that Tregs enhance the differentiation and function of tissue stem cells by modulating local inflammatory responses, thereby improving the tissue repair efficiency (67). For instance, after skin damage, hair follicle stem cells are recruited to the damaged area and differentiate into epithelial cells to rebuild the skin barrier (125). In cardiovascular injury, Tregs have been found to promote the proliferation and differentiation of cardiac stem cells, thereby improving cardiac function (110).

Conversely, Tregs are also regulated by non-immune cells. Mesenchymal stem cells can activate Tregs through ICOS-ICOSL interactions, enabling Tregs to suppress the activity of ILC2s, which play a role in controlling type 2 immune responses mediated by the allergic cytokines IL-13, IL-5, and IL-9 (94). These mechanisms accordingly indicate that tissue Tregs not only play essential roles in immune regulation but, via their interactions with non-immune cells, also facilitate tissue repair and regeneration.

### 3.3 In fibrosis

As a consequence of defective repair, chronic inflammatory responses can lead to fibrosis (126–128). By suppressing inflammation and interacting with different types of non-immune cell, tissue Tregs can contribute to the regulation of fibrotic processes (**Table 2**), a key aspect of which is the functional regulation of fibroblasts, which play central roles in both wound healing and fibrosis. Research has shown that tissue Tregs promote fibroblasts proliferation and activation by secreting AREG (14, 149). Although this process is conducive to developing an extracellular matrix and tissue regeneration, the excessive proliferation and activation of fibroblasts often lead to tissue fibrosis. By suppressing inflammation and attenuating excessive fibroblast activity, tissue Tregs can contribute to preventing fibrosis and scar formation (150). These bidirectional interactions influence both the immune status of the local microenvironment and the overall quality of tissue healing and functional recovery. Consequently, studying the interactions between tissue Tregs and fibroblasts could provide valuable insights for the development of new treatment strategies designed to enhance tissue repair and prevent fibrosis.

**Table 2:** The dual roles of tissue Tregs in fibrosis.

Tissue	Interacting cells (Immune/non-immune cells)	Mechanisms	Reference	Effect
Lung Tregs	Macrophages	Tff1^+^ Tregs inhibit the pro-inflammatory features of macrophages	(129)	Inhibit fibrosis
		TIM-3^+^ Tregs regulate macrophage polarization	(130)	Inhibit fibrosis
	Th	Promote the conversion from Th1 to Th2	(131)	Promote fibrosis
	CD103^low^ Trm	Suppression of inflammatory responses	(132)	Inhibit fibrosis
	Lung fibroblasts	Secrete PDGF and TGF-β, promote the accumulation of lung fibroblasts	(133, 134)	Promote fibrosis
		Reduce the recruitment of fibroblasts by reducing the signaling of the CXCL12/CXCR4 axis and suppressing CXCL10 and CCL2	(131, 135, 136)	Inhibit fibrosis
	Lung epithelial cells	Promote the proliferation, activation, and EMT of alveolar epithelial cells	(137)	Promote fibrosis
Skin Tregs	Th	Produce pro-fibrotic Th2 cytokines	(138)	Promote fibrosis
		GATA-3 expression on skin Tregs inhibits Th2 polarization	(139)	Inhibit fibrosis
	Dermal fibroblasts	Secretion of TGF-β	(140)	Promote fibrosis
		Activate the AREG–EGFR–MEK signaling axis	(141)	Promote fibrosis
		Low expression level of TGF-β	(142)	Inhibit fibrosis
Liver Tregs	Monocytes/Macrophages	Inhibit the activation of Ly-6C^high^ inflammatory monocytes/macrophages	(15)	Inhibit fibrosis
	Th2	Suppress the activation and expansion of Th2 cells that produce IL-4	(15)	Inhibit fibrosis
	HSCs	Activate HSCs via the TGF-β pathway and by increasing IL-8 levels	(143)	Promote fibrosis
		Protect HSCs from NK cell attack by inhibiting NK cell degranulation via IL-8, TGF-β1, and CTLA-4 signaling pathways	(144)	Promote fibrosis
		ST2^+^Tregs promote the activation of HSCs by secreting AREG	(14)	Promote fibrosis
		Inhibit HSC activation by suppressing MCP-1 and preventing CD4^+^ T cells from secreting IFN-γ	(145)	Inhibit fibrosis
	KCs	Inhibit the secretion of MMPs by KCs *in vivo* via the TGF-β pathway	(146)	Inhibit fibrosis
	hAMSCs	Enhance the tissue repair capacity of hAMSCs	(147)	Inhibit fibrosis
Cardiac Tregs	Fibroblasts	Produce SPARC, increase the production of collagen III in fibroblasts	(148)	Inhibit fibrosis

Tff1, trefoil factor family 1; TIM-3, mucin domain-containing protein 3; Th, T helper cells; Trm, resident memory T cells; PDGF, platelet-derived growth factor; TGF-β, transforming growth factor-β; CXCL, chemokine C-X-C motif ligand; CXCR, C-X-C motif receptor; CCL, chemoattractant chemokine ligand; EMT, epithelial-mesenchymal transition; GATA-3, GATA binding protein 3; EGFR, epidermal growth factor receptor; MEK, mitogen-activated protein kinase/extracellular receptor-stimulated kinase; HSCs, hepatic stellate cells; MCP-1, monocyte chemoattractant protein-1; KCs, Kupffer cells; hAMSCs, human amniotic mesenchymal stem cells; SPARC, secreted protein acidic and rich in cysteine; α-SMA, anti-smooth muscle antibody; MMP, matrix metalloproteinase.

## 4 The roles of tissue Tregs in tissue repair and fibrosis

In this section, we discussed the specific roles of tissue Tregs in tissue repair and fibrosis across various pathological contexts, focusing on tissues such as the lung, skin, bone, skeletal muscle, liver, heart, intestine, and brain.

### 4.1 Lung Tregs

#### 4.1.1 The role of lung Tregs in acute lung injury

**Figure 1:**
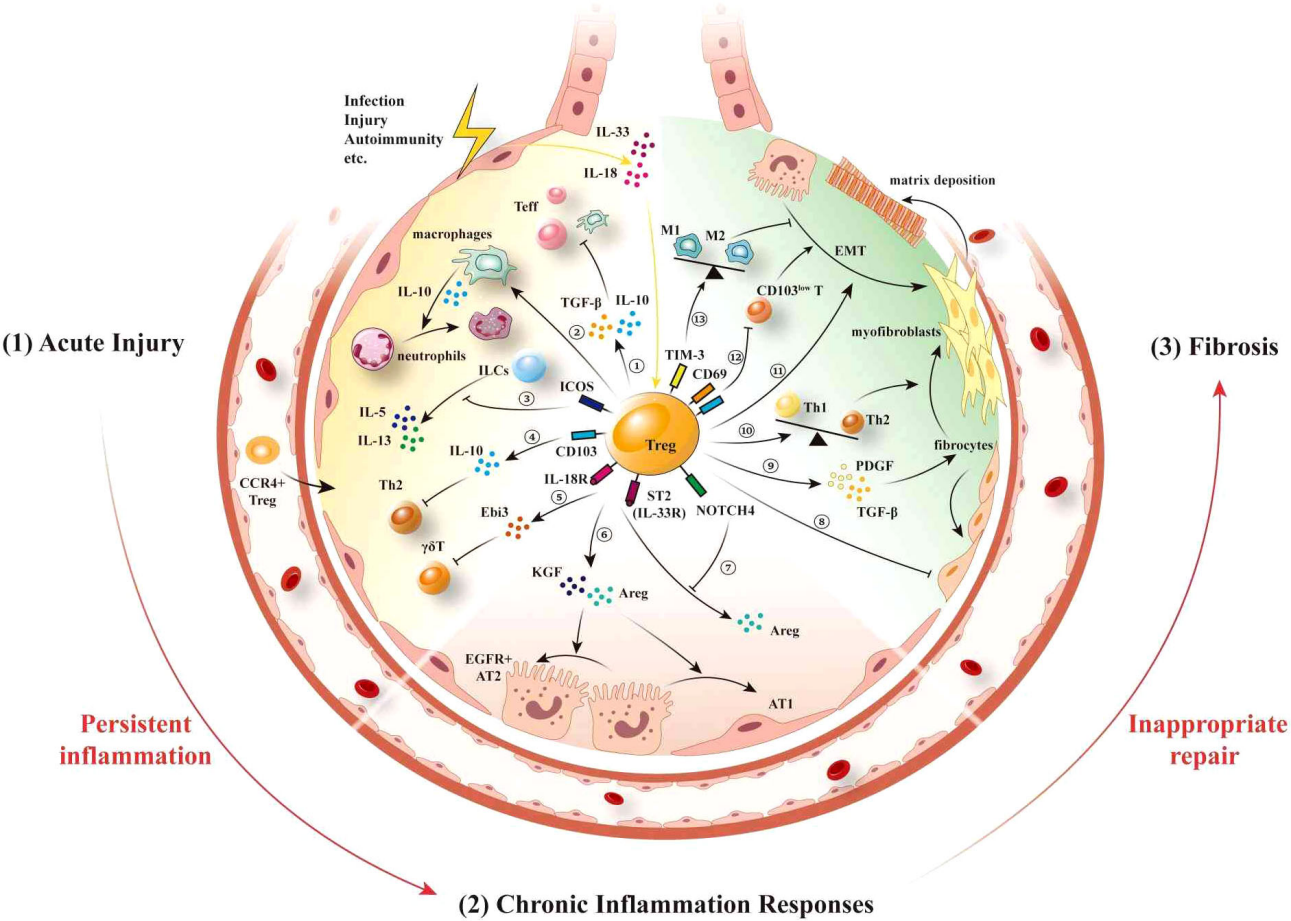
Lung Tregs interacts with other immune/non-immune cells following injury. After suffering injury, lung epithelial cells release the “alarmins” IL-18 and IL-33, thereby promoting the migration of inflammatory cells to the lungs, leading to pulmonary inflammation. Expression of CCR4 stimulates the recruitment of Tregs to the lungs (1). During the acute phase of injury, neutrophils and macrophages are initially recruited to participate in the acute inflammatory response, further causing damage to the lung epithelium. ① Lung Tregs secrete the inhibitory factors IL-10 and TGF-β to suppress the proliferation and activation of other immune cells. ② Lung Tregs promote neutrophil apoptosis mediated by macrophage, and ③ also suppress the activation of ILC2s by directly inducing ICOS, which inhibits the production of IL-5 and IL-13 from ILCs. ④ CD103^+^ Treg-expressed IL-10 suppresses Th2-type inflammatory responses. ⑤ IL-18 and IL-33 activate IL-18R and ST2 on Tregs, thereby inhibiting the function of γδT cells by via the secretion of Ebi3 (2). During the chronic inflammatory response phase, ⑥ ST2^+^ Tregs secrete AREG and KGF, thereby promoting the proliferation and differentiation of AT2 cells. ⑦ By expressing vimentin and NOTCH4, Tregs respectively inhibit the secretion of AREG mediated by the activation of IL-18R and ST2 (3). At the fibrotic stage, ⑧ lung Tregs can control the recruitment of fibroblasts and alleviate pulmonary fibrosis. ⑨ Tregs promote fibroblast proliferation and their transformation to myofibroblasts and matrix deposition by secreting PDGF and TGF-β. ⑩ Tregs promote Th2 polarization, which in turn promotes fibrosis. ⑪ Tregs promote EMT, whereas ⑫ CD69^high^CD103^high^ Tregs in the lung inhibit fibrosis caused by CD103^low^ resident memory T cells. ⑬ TIM-3^+^ Tregs contribute to reducing pneumonia and lung injury by regulating macrophage polarization. IL, interleukin; CCR, chemokine (C-C motif) receptor; TGF-β, transforming growth factor-β; ILC2, type 2 innate lymphoid cells; ICOS, inducing co-stimulation; ST2, growth stimulation expressed gene 2; Ebi3, Epstein-Barr virus-induced gene 3; Th, T helper cells; AREG, amphiregulin; KGF, keratinocyte growth factor; AT2, alveolar type II cells; PDGF, platelet-derived growth factor; EMT, epithelial-mesenchymal transition; CD, cluster of differentiation; TIM-3, mucin domain-containing protein 3.

Inflammatory responses trigger the local infiltration of lung Tregs (151), which are instrumental in resolving lung inflammation and promoting tissue recovery in cases of acute respiratory infections and acute lung injury (**Figure 1**). In this regard, it has been established that the expression of CCR4 is vital for initiating the lung-specific recruitment of Tregs, and it has been demonstrated that a deficiency in CCR4 is associated with limited lung trafficking and an inability to suppress lung inflammation effectively (32). In the context of acute lung injury (ALI), the release of local inflammatory factors such as IL-6 and TNF-α can also promote the activation and proliferation of Tregs, thereby enhancing their infiltration into lung tissue (152). Subsequent to their recruitment or expansion in the lungs, Tregs contribute to maintaining homeostasis by interacting with different immune cell types (82, 92). In models of acute lung injury, the recruitment of diverse types of immune cells, including neutrophils and macrophages, along with inflammatory mediator release, leads to endothelial damage (153, 154). Tregs regulate immune responses and suppress inflammatory through various mechanisms. They inhibit the activation and chemotaxis of neutrophils by secreting TGF-β and IL-10, reducing their aggregation and activity at inflammatory sites (89). Furthermore, Tregs reduce neutrophil-mediated inflammation by downregulating pro-inflammatory cytokines such as IFN-γ. This mechanism has been validated in various inflammatory diseases, including ALI models, where Treg deficiency leads to excessive neutrophil activation and exacerbated inflammation (90). Tregs also promote the transformation of macrophages from M1 to M2 phenotype by enhancing IL-10 secretion, thereby alleviating inflammation in ALI (155, 156). Moreover, Tregs enhance macrophage anti-inflammatory functions via IL-13 secretion, which stimulates macrophages to produce IL-10. This IL-10 induces autocrine-paracrine signaling of Vav1 in macrophages and activates Rac1 to promote macrophage efferocytosis (153). Through these mechanisms, Tregs enhance the phagocytic function of macrophages, enhancing apoptotic cell clearance, and preventing necrosis and subsequent inflammation.

Certain subsets of lung Tregs have been demonstrated to play significant roles in lung injury. For example, IL-33-mediated ST2^+^ Tregs have been found to secrete IL-13 to control inflammatory response following lung injury (97), whereas Faustino et al. (96) found that by promoting the expression of Epstein-Barr virus-induced gene 3 (*Ebi3*), a component of IL-35, ST2^+^ Tregs can act as early negative regulators of innate γδ T cells, thus reducing allergen-induced lung inflammation. Furthermore, lung Tregs expressing tumor necrosis factor receptor (TNFR2) are recognized as a suppressive and proliferative subset (95). In lungs infected with pneumococcus, TNFR2^+^ Tregs inhibit γδ T cells by reducing their secretion of the pro-inflammatory cytokine IL-17A, thereby preventing excessive pulmonary inflammation (157). Moreover, Tregs expressing CD103 represent a unique subset that specifically suppress Th2 responses, driving the resolution of Th2-mediated allergic airway inflammation via elevated levels of IL-10 expression (98). On the basis of these “classical” immunosuppressive mechanisms, lung Tregs thus contributes to mediating the resolution of inflammation during the acute injury phase.

#### 4.1.2 The role of lung Tregs in chronic inflammatory responses

Although antigen-specific lung Tregs generated in response to acute injury dampen the immune response to pathogens and limit inflammation-related damage, they may also contribute to the chronic persistence of inflammation. In ongoing inflammatory situations, lung Tregs not only limit inflammation but also interact with diverse non-immune cells via direct interactions and indirect effects via other immune/non-immune cells to promote tissue repair and regeneration (**Figure 1**). Moreover, by promoting neutrophil infiltration and upregulating the release of TGF-β1, CD103^+^ lung Tregs have also been demonstrated to promote AT2 cell proliferation in a CD103-dependent manner (100, 101, 158). In contrast, in response to the release of IL-18 and IL-33 from damaged tissues, lung Tregs produce significant amounts of AREG (159), a cytokine that facilitates tissue repair by mediating EGFR-induced inhibition of the pro-apoptotic effects of TNF-α on AT2 cells (80). AREG also stimulates the proliferation and differentiation of AT2 cells (158), as exemplified by Treg-derived AREG stimulation of a population of Col14a1^+^EGFR^+^ mesenchymal cells, which mediates the regeneration of AT2 cells during influenza-induced lung injury in mice (87). However, Tregs also express certain inhibitory factors that can contribute to diminishing the activity of AREG, as illustrated by the expression of NOTCH4 on Tregs, which dynamically suppresses AREG-dependent tissue repair, leading to elevated levels of pulmonary inflammation (160). Similarly, the type III intermediate filament protein vimentin has been established to suppress the IL18R- mediated increase in AREG, thereby impairing lung tissue repair (119). This dynamic regulation of AREG accordingly highlights its therapeutic implications for related diseases. Additionally, by secreting KGF, lung Tregs have been demonstrated to promote AT2 cell proliferation (86). Moreover, the repair of alveolar endothelial cells is necessary for restoring gas exchange following lung injury. It has been demonstrated in mice that lung Tregs are essential for lung angiogenesis (99), although the precise underlying mechanisms have yet to be established.

#### 4.1.3 The role of lung Tregs in fibrosis

The proliferation and activation of alveolar epithelial cells promote tissue repair through regeneration (161). In contrast, tissue repair based on the activation of fibroblasts is often considered detrimental because it significantly contributes to fibrosis and organ dysfunction (162). Moreover, the proportion and quantity of lung Tregs produced during pulmonary fibrosis can either increase or decrease (133), thereby complicating the elucidation of the specific roles played by lung Tregs in this condition. For example, the lungs and blood of patients with connective tissue disease-associated interstitial pneumonia (CTD-IP) are typically characterized by elevated levels of cytotoxic T cells and lower levels of Tregs (163). In contrast, elevated levels of Tregs have been detected in the blood and lungs of patients with advanced fibrosis (164). A commonly used model for studying pulmonary fibrosis in mice is bleomycin (BLM)-induced acute lung inflammation (165), which subsequently leads to fibrosis, and the findings of studies using this model have revealed that the role of Tregs in the pathogenesis of pulmonary fibrosis differs depending on the stage of the disease (166). Moreover, studies that have involved the transfer or depletion of Tregs, indicate that these cells can have both protective or harmful effects during different phases of BLM-induced lung injury (167, 168). In summary, there remain considerable challenges with respect to determining whether the observed changes in lung Tregs during pulmonary fibrosis are a “cause” or a “consequence” of this disorder; that is, whether lung Tregs drive the progression of pulmonary fibrosis or react in response to counteract fibrosis. Nevertheless, research to date tends to indicate that lung Tregs play a dual role in both preventing and contributing to the development of pulmonary fibrosis (131) (**Figure 1**).

Lung Tregs contribute to pulmonary fibrosis via multiple mechanisms, among which they play roles in influencing the Th1/Th2 balance, generating a fibrosis-conducive cytokine environment, promoting epithelial-mesenchymal transition (EMT), and facilitating the proliferation and differentiation of fibroblasts, as well as collagen deposition. For example, in a mouse model of silica-induced pulmonary fibrosis, the depletion of lung Tregs has been found to promote an enhanced Th1 response and disrupt the Th1/Th2 balance, thereby resulting in a shift toward a Th2 phenotype (131). Lung Tregs have also been established to promote the progression of pulmonary fibrosis by secreting factors such as platelet-derived growth factor (PDGF) and TGF-β, specifically targeting lung fibroblasts. TGF-β has been identified as a key mediator in the fibrotic process, inducing the proliferation of fibroblasts and their subsequent transformation to myofibroblasts (134). Similarly, the PDGF-induced promotion of fibroblast proliferation contributes to an excessive production of extracellular matrix components (133). Furthermore, in cases of radiation-induced pulmonary fibrosis, it has been established that lung Tregs facilitate the accumulation of fibrocytes in the irradiated lungs, and in epithelial cells promote β-catenin-mediated EMT (137). Collectively, the crosstalk among lung Tregs, other infiltrating T cells, epithelial cells, and fibroblasts, contribute to the activation of myofibroblasts, thereby promoting the deposition of collagen, and ultimately leading to the destruction of the typical lung structure.

Contrastingly, lung Tregs can also play a protective role in pulmonary fibrosis. Lung Tregs can help prevent fibrosis by resolving inflammatory responses. An example is the activation of the AhR signaling pathway, which boosts Tregs numbers and reduces inflammatory T cell subsets, thereby decreasing pulmonary fibrosis in the BLM model (169). Specifically, CD69^high^CD103^high^ Tregs represent a protective subset in lung inflammation and fibrosis. In a fungal antigen-induced pulmonary fibrosis model, CD103^low^ resident memory T cells selectively express profibrotic cytokine genes *Il5* and *Il1*. In contrast, CD69^high^CD103^high^Foxp3^+^ Tregs exhibit elevated expression of *Itgae* and *Foxp3*, effectively suppressing the profibrotic and inflammatory responses driven by CD103^low^ resident memory T cells (132). Lung Tregs expressing trefoil factor family 1 (Tff1) can prevent the worsening of BLM-induced pulmonary fibrosis. They achieve this by inhibiting macrophage pro-inflammatory responses and reducing the quantity and activity of inflammatory myeloid cells (129). In addition, lung Tregs inhibit fibroblast proliferation, helping to prevent the progression of pulmonary fibrosis. It has been demonstrated that by reducing chemokine C-X-C motif ligand 12 (CXCL12) and C-X-C motif receptor 4 (CXCR4) signaling (135), as well as suppressing CXCL10 (131) and CCL2 (136), lung Tregs can play roles in controlling the recruitment of fibroblasts, thereby alleviating pulmonary fibrosis (135).

### 4.2 Skin Tregs

#### 4.2.1 The role of skin Tregs in acute tissue injury

Skin Tregs facilitate early wound healing after acute injury by recruiting monocytes and macrophages to injury sites. Single-cell sequencing reveals that injury triggers preferential expression of integrin αvβ8 in skin Tregs, which activates latent TGF-β, enhancing CXCL5 production and neutrophil recruitment (170). Additionally, skin Tregs interact with keratinocytes through Jag1-Notch signaling, inducing the release of chemokines by keratinocytes that attracts monocytes and neutrophils to the site of injury (103). Although these mechanisms may slightly delay epidermal regeneration, they provide essential protection against infection, demonstrating the important role of Tregs in acute tissue damage.

Conversely, skin Tregs also prevent excessive immune responses by suppressing immune cells. They not only regulate immune responses by suppressing the activity of Teffs, but also promote the polarization of macrophages towards the M2 phenotype (171). In addition to mitigating inflammation, EGFR signaling and CD103 expression support the migration and survival of Tregs at injury sites (102, 172). The ligand AREG for EGFR can be expressed by Tregs infiltrating the injured tissue (159), while the ligand E-cadherin for CD103 is mainly expressed by epithelial cells (173). EGFR expression on Tregs reduces IFN-γ production and limits the accumulation of pro-inflammatory macrophages (102). Studies have shown that the specific removal of EGFR^+^ skin Tregs results in delayed re-epithelialization and altered rates of wound closure (174), underscoring their essential roles in maintaining immune balance and wound healing. Moreover, CD103^+^ Tregs suppress inflammation by downregulating the pro-inflammatory function of dendritic cells (DCs) through contact-dependent mechanisms, such as CTLA-4-CD80/86 and PD-L1/PD-1 axis (175).

#### 4.2.2 The role of skin Tregs in chronic inflammatory responses

Skin Tregs facilitate the regeneration and repair of epithelial cells during the chronic inflammation phase through various mechanisms. The skin contains a substantial number of type 2 polarized Tregs that are programmed by Th2-related transcription factors, including GATA-3 and IRF4, which are important for tissue repair (176). GATA-3^+^ Tregs in the skin have been established to express receptors for alarm signals, such as TSLP, IL-33, and IL-18, which are released during tissue damage, thereby enabling these Tregs to sense local injuries (177). Similar to lung Tregs, skin Tregs also participate in tissue repair by directly secreting different repair mediators, among which, both IL-18 and IL-33 can stimulate the expansion of skin Tregs that produce the repair-associated cytokine AREG in the absence of TCR stimulation (174). In addition to promoting the growth of keratinocytes (104), AREG also contributes to the restoration of vascular integrity by enhancing TGF-β activation in pericytes. In a model of ultraviolet B radiation (UVB)-induced skin damage, healing-associated skin Tregs have been observed to proliferate in response to UVB exposure and secrete proenkephalin (PENK), a precursor of opioid-like substances, that promotes the growth of epidermal keratinocytes (105). Furthermore, by activating progenitor cells, skin Tregs can facilitate the regeneration of skin (71). For example, research has shown that Tregs promote the differentiation of hair follicle stem cells (HFSCs) into epithelial cells during the skin barrier repair process (106).

#### 4.2.3 The role of skin Tregs in fibrosis

The fibrosis of skin is a defining characteristic of systemic sclerosis (SSc) (178, 179), and research in this regard has revealed increases in the levels of Tregs in peripheral blood and skin lesions during the inflammatory and fibrotic phases of the disease (180). However, these Tregs are often dysfunctional and have a reduced suppressive capacity (181), and the findings of some studies have indicated that compared with healthy skin or psoriatic skin lesions, skin Tregs are less prevalent in SSc, and that this reduction is correlated with reductions in the levels of TGF-β and IL-10 (182). The findings of a further study have indicated that compared with late-stage SSc patients and healthy controls, skin Tregs are more numerous in the skin epidermis and dermis of early SSc patients (183), whereas in patients with limited and diffuse SSc, the Tregs in skin lesions have been found to produce pro-fibrotic Th2 cytokines, such as IL-13 and IL-4 (138). Consequently, these dysfunctional skin Tregs may contribute to an exacerbation of the disease. Collectively, the findings of these studies provide evidence of an association between the quantitative reduction and/or qualitative dysfunction of skin Tregs and the occurrence SSc. However, there is currently a lack of consensus in this regard.

By interacting with dermal fibroblasts, skin Tregs contribute to the occurrence of pathological skin fibrosis (138). These Tregs secrete TGF-β, a well-known profibrotic factor (140). Moreover, while AREG promotes tissue repair, it can also promote fibrosis. It has been established that the AREG-EGFR-MEK (mitogen-activated protein kinase/extracellular receptor-stimulated kinase) signaling axis plays a central role in mediating the development of skin fibrosis. For example, using models of BLM-induced skin fibrosis, Zhang et al. (141) have shown that AREG is upregulated throughout the fibrogenesis process and is associated with an enhanced proliferation of dermal cells. Conversely, dermal cells proliferation induced by BLM does not occur in mice that lack the AREG gene. Moreover, trametinib, which inhibits MEK (a downstream effector of AREG), has proven effective in preventing skin fibrosis in models induced by BLM.

Skin Tregs may also contribute to a reduction in fibrosis. In this regard, although skin Tregs secrete TGF-β, the amounts are relatively low, but may still potentially serve as a “TGF-β reservoir” that inhibits fibroblast activation (184). In animal models of SSc (142), both the acute depletion and chronic reduction in skin Tregs lead to the spontaneous activation of skin fibroblasts, along with an increase in the expression of pro-fibrotic genes, and subsequent dermal fibrosis, thereby highlighting their key roles in the pathology of skin diseases. Additionally, skin Tregs have been shown to be characterized by elevated levels of GATA-3 expression, which is assumed to be associated with Th2 polarization (177). Conversely, in the absence of GATA-3, there are larger numbers of Th2 cells and increases in fibroblast activation, thus tending to indicate that the GATA-3 in skin Tregs has certain beneficial effects that contribute to the prevention of skin fibrosis (139, 182).

### 4.3 The functions of other Tregs in tissue repair and fibrosis

In addition to the lungs and skin, the findings of numerous studies have provided evidence to indicate that by interacting with immune/non-immune cells, tissue Tregs play roles in the repair and fibrosis of other tissues.

#### 4.3.1 Liver Tregs

In the liver, Tregs have been established to play roles in the response to liver injury and in managing chronic inflammation. In the acute phase of liver injury, immune cells trigger inflammation, thereby leading to a rapid apoptosis-induced reduction in the population of liver Helios^+^ Tregs, and this contributes to the progression of inflammation and tissue damage (185). During the healing phase, inflammation subsides, wound healing is initiated, and immune homeostasis is restored. Hepatic stellate cells (HSCs) promote regeneration of the Helios^+^ Tregs subset via matrix metalloproteinase (MMP) 9/13-dependent TGF-β activation, which is essential for terminating inflammation and facilitating wound healing (185), thus, emphasizes the important role played by Helios^+^ Tregs as a “repair” subset in liver injury.

Liver Tregs exhibit a dual role in the onset and progression of liver fibrosis across various liver injury models. In the context of non-alcoholic steatohepatitis, liver ST2^+^ Tregs significantly contribute to liver tissue repair and fibrosis regulation by secreting AREG (14). Conversely, in carbon tetrachloride (CCl4)-induced liver inflammation and fibrosis, liver Tregs expand preferentially, helping to prevent fibrosis by limiting the abnormal activation of pre-fibrotic immune cells, such as Th2 cells and Ly-6C^high^ inflammatory monocytes/macrophages (15). Furthermore, liver Tregs-expressed CD39 has been demonstrated to be associated with the suppression of the CD8^+^ T cell proliferation and their production of TNF-α and osteopontin, thereby alleviating biliary fibrosis (186).

Recent studies have highlighted the impact of liver Tregs interaction with various non-immune cells in liver fibrosis progression. First, liver Tregs have been shown to directly activate hepatic stellate cells (HSCs), which can differentiate into myofibroblast-like cells, producing extracellular matrix and cytokines (187) that promote fibrosis (188). Conversely, activated HSCs secrete matrix metalloproteinases (MMPs) that degrade the extracellular matrix, potentially inhibiting fibrosis (189–191). Second, natural killer (NK) cells regulate liver fibrosis by targeting activated HSCs (192), while Tregs can indirectly modulate HSC activity by suppressing NK cells (144) through direct cell contact (CTLA-4 signaling) (193) and cytokine release (IL-8 and TGF-β) (194). Therefore, by modulating the interaction between NK cells and HSCs, liver Tregs can alter the progression of liver fibrosis. Additionally, liver Tregs can prevent HSC activation by suppressing monocyte chemoattractant protein-1 (MCP-1) and inhibiting the IFN-γ-secretory activity of CD4^+^ T cells (146), thereby conferring liver protection (143). In summary, the regulatory mechanisms employed by liver Tregs on HSCs are crucial to the progression of liver fibrosis.

In turn, HSCs can mutually influence liver Tregs by promoting an IL-2-dependent increase in the numbers of these Tregs (186). *In vitro* experiments have revealed that a proliferation of allogeneic Tregs promoted by mature HSCs is dependent on both dose and cell contact, and enhances the Tregs-mediated suppression of Teff proliferation (186). Furthermore, by modulating the balance between Treg and Th17 cell responses, it has been demonstrated that the transfer of HSC-activated Tregs can contribute to a significant reduction in liver injury in animal models of autoimmune hepatitis (AIH). This highlights the importance of HSC regulation on Tregs in the pathology of liver injury.

In addition to HSCs, liver Tregs have been shown to inhibit the secretion of MMPs by Kupffer cells *in vivo* via the TGF-β pathway (146), thereby preventing fibrosis regression. Moreover, these Tregs can modulate human amniotic mesenchymal stem cells (hAMSCs) to enhance their tissue repair functions via TGF-β and indoleamine 2,3-dioxygenase, thereby promoting the hAMSC-mediated inhibition of fibrosis (147).

#### 4.3.2 Cardiac Tregs

Cardiac Tregs play key roles in the healing process following various cardiac injury diseases, such as myocardial infarction (MI) (107). After tissue damage, cardiac Tregs initially interact with immune cells to control local inflammation. Cardiac Tregs suppress the pro-inflammatory M1 phenotype of macrophages by secreting IL-10 and TGF-β, promoting their transformation into anti-inflammatory/repair M2 phenotypes. In a mouse model of myocardial ischemia-reperfusion, the absence of Tregs leads to sustained secretion of TNF-α and IL-6 from macrophages, exacerbating myocardial injury (195). Additionally, Tregs suppress macrophage CD80/CD86 co-stimulatory signals through a CTLA-4-dependent pathway, limiting excessive inflammatory responses (107). Cardiac Tregs also promote neutrophil apoptosis by secreting lipoxin A4 (LXA4) and inhibit the formation of neutrophil extracellular traps (NETs). In a myocardial infarction-induced fibrosis model, Treg-deficient mice show prolonged neutrophil infiltration and abnormal collagen deposition (108). Tregs inhibit the differentiation of Th17 cells through cell-cell contact, such as the PD-1/PD-L1 pathway, thereby reducing IL-17-mediated myocardial fibrosis. They further regulate CD8^+^ T cell activation to prevent toxic damage to surviving cardiomyocytes (196).

Moreover, cardiac Tregs directly interact with parenchymal cells, including cardiomyocytes (CMs) and endothelial cells. ATP released by damaged CMs activates the P2X7 receptor on Tregs, enhancing their immunosuppressive function. Conversely, IGF-1 secreted by Tregs inhibits CMs apoptosis through the PI3K/Akt pathway, promoting their survival (197). Additionally, Tregs promote the regeneration of CMs by secreting regenerative factors including CCL24 (which stimulates proliferation through ERK1/2 signaling), AREG (an EGFR pathway activator), and GAS6 (a mediator of efferocytic clearance) (109). Tregs also promote endothelial cell proliferation and angiogenesis by secreting VEGF-A while inhibiting ICAM-1 expression, which reduces leukocyte adhesion and vascular leakage. In atherosclerosis models, Tregs expansion significantly improves endothelial function (197).

Fibroblasts play essential roles in preserving the integrity of the injured heart. Their activation facilitates effective repair and stable collagen deposition following cardiac injury (110). Within the infarct area of MI, the accumulation of fibroblasts in the hearts of mice has been found to reduce the risk of cardiac rupture after MI, primarily by enhancing collagen III production by fibroblasts (148). However, an excessive activation of fibroblasts or insufficient apoptosis of myofibroblasts following cardiac injury often contributes to poor repair-associated responses. Tregs regulate fibroblast activity and exhibit a dual role depending on the disease stage. During the acute repair phase, Tregs suppress fibroblast differentiation into myofibroblasts by secreting IL-10, thereby mitigating excessive collagen deposition. In a mouse model of myocarditis, the adoptive transfer of Tregs has been shown to lower the activation of the TGF-β/Smad3 pathway (107), reflecting their protective effect against fibrosis. In the chronic fibrosis phase, Tregs also exhibit a dual role in regulating fibrosis. In models of long-term stress overload, Tregs promote fibroblast proliferation and extracellular matrix (ECM) remodeling by secreting AREG, which activates the EGFR/ERK pathway, potentially leading to excessive fibroblast activation and aggravated fibrosis (197). However, in diabetic cardiomyopathy fibrosis, Tregs improve fibrosis by competitively absorbing glutamine, inhibiting fibroblast mitochondrial oxidative phosphorylation, and reducing their anabolic activity (108). A unique population of ST2^+^ Tregs has been identified that produces secreted protein acidic and rich in cysteine (SPARC) (148). *In vitro* studies have indicated that co-culturing fibroblasts with cardiac Tregs expressing SPARC inhibits the excessive activation of fibroblasts, which accordingly provide evidence to indicate a protective role of cardiac Tregs in fibrosis.

However, given the complexity of the multiple interactions between cardiac Tregs and the surrounding tissue cells, further studies are necessary to better understand the roles played by this Treg subset.

#### 4.3.3 Muscle and bone Tregs

Studies show that Tregs rapidly migrate to skeletal muscle injury sites, driven by T cell receptor (TCR) signaling and IL-33 released by bone marrow-derived mesenchymal stem cells (MuSCs) (50, 62, 198, 199). At the injury site, Tregs release anti-inflammatory factors such as IL-10 and TGF-β, which help modulate the inflammatory response. The upregulation of TGF-β also enhances Treg functionality and promotes their migration to the injury site (200). Furthermore, Tregs promote macrophage polarization towards the anti-inflammatory M2 phenotype (201). By secreting growth factors like AREG (111), Tregs activate and expand muscle progenitor cells (MPCs), aiding in their differentiation into muscle cells.

Tregs play a crucial role in bone healing, a complex process that requires coordinated interactions among osteoblasts, osteoclasts, and immune cells. The dynamics of Tregs, including their numbers and functionality, are critical for healing outcomes, particularly in vulnerable populations like diabetic and elderly patients (112). Tregs foster a conducive microenvironment for bone healing by secreting anti-inflammatory cytokines, including IL-10 and TGF-β (202, 203), and directly enhancing osteoblast proliferation and differentiation (204). During fracture healing, Tregs secrete amino acids and growth factors, such as amphiregulin (AREG), to stimulate the proliferation and differentiation of osteoblast progenitor cells, thereby promoting bone formation (203).

The activity of Tregs is related to bone damage and synovial fibrosis in rheumatoid arthritis (RA). A deficiency in functional Tregs results in excessive osteoclast activation and further bone destruction (205). In RA patients, the numbers and functions of Tregs are frequently compromised, resulting in a loss of immune tolerance and heightened autoimmune responses. This not only exacerbates bone destruction but also worsens synovial fibrosis (206). In synovial fibrosis associated with RA, Tregs help regulate immune responses and exert immunosuppressive effects that inhibit Teff activation, thereby alleviating synovial inflammation and fibrosis (207). In addition, Tregs significantly influence the activity of synovial fibroblasts (SFs). Induced Treg cells (iTregs) have demonstrated inhibitory effects on SFs through cytokines such as IL-10 and TGF-β, which inhibit SF proliferation and inflammatory responses (208).

#### 4.3.4 Intestinal Tregs

Intestinal Tregs have been established to promote tissue repair and contribute to maintaining the integrity of the gut epithelial barrier. They secrete anti-inflammatory factor IL-10 to suppress excessive inflammation, promote the regeneration of intestinal epithelial cells (IECs), and maintain barrier integrity (209). IL-10 alleviates endoplasmic reticulum stress and protects the epithelial barrier by suppressing IECs fucosylation and Fas-mediated apoptosis (210).

Intestinal Tregs also express repair-related markers, such as AREG and ST2, with the IL-33/ST2 signaling pathway drives their accumulation in the intestine, which alleviates colitis injury by enhancing Foxp3 expression (113, 211). Knockdown of the IL-33/ST2 pathway aggravates tissue damage. Studies suggest that although repair subsets of intestinal Tregs increase among HIV-infected individuals, defects in AREG secretion leads to impaired epithelial repair (212), highlighting the importance of AREG in gut epithelial restoration. Additionally, within the gut mucosa, human CD161^+^ Tregs, which are regulated by retinoic acid, have been demonstrated to facilitate wound repair (213). Furthermore, by contributing to the renewal of epithelial stem cells, intestinal Tregs have been found to promote homeostasis in intestinal epithelial cells (113). Intestinal Tregs can enhance crypt stem cell activity and promote epithelial renewal through the Wnt/β-catenin pathway (214). Studies have shown that the absence of Tregs in colitis models is associated with a decline in crypt stem cell function. In the intestinal crypts, Tregs also maintain the stem cell microenvironment homeostasis by regulating the levels of local cytokines (such as IL-22), thereby maintaining the balance between stem cell proliferation and differentiation (215).

#### 4.3.5 Brain Tregs

Brain Tregs play essential roles in the repair of brain tissues following neuroinflammation and injury (216). Accumulating evidence indicates that these Tregs play a protective role during the acute phase of stroke and contribute to recovery in the chronic phase. Brain Tregs target a range of cell types, including immune and central nervous system cells, on which they have beneficial effects via their influence on intercellular interactions and the release of soluble factors (115, 217). For example, these Tregs secrete cytokines such as AREG, which modulate astrocyte responses and thereby contribute to reducing neurological damage, and also express neuron-specific genes, such as the serotonin receptor (Htr7), and respond to serotonin, which leads to an increase in Tregs numbers and an amelioration of neurological symptoms (84).

Collectively, the findings of these studies highlight the pivotal roles played by tissue Tregs in immune regulation and tissue repair among different organs, and thus, gaining a more comprehensive understanding of the mechanisms underlying the activity of these cells in different immune microenvironments will be essential for developing effective therapies for the treatment of fibrosis.

## 5 Conclusion

Tissue Tregs have been established to play multiple complex roles in injured and fibrotic tissues. Recent research has provided compelling evidence to indicate their essential function in promoting tissue repair. During the acute phase of injury, by interacting with other immune cells, tissue Tregs primarily contribute to the control of inflammation, whereas in the chronic phase of inflammation, they extend their role beyond immune modulation by engaging with non-immune cells to promote tissue repair. However, the precise role of Tregs in fibrosis has sparked considerable debate, which can partially be explained by their dual regulatory effects on fibroblasts. It has also been found that different subsets of tissue Tregs that express distinct suites of functional molecules may have certain tissue-specific roles, thereby emphasizing the need to study the diversity of tissue Tregs and the potential for targeted therapy via molecular regulation. Future research should focus on adjusting tissue Tregs in their local environments to balance their roles in tissue repair and the prevention of fibrosis. In summary, a comprehensive understanding of the regulatory functions of tissue Tregs in tissue repair and fibrosis, as well as their specific activities in the context of differing physiological and pathological states, will provide vital insights and practical guidance for future research and clinical applications.
